# Individual and Community Level Risk-Factors for Alcohol Use Disorder among Conflict-Affected Persons in Georgia

**DOI:** 10.1371/journal.pone.0098299

**Published:** 2014-05-27

**Authors:** Bayard Roberts, Adrianna Murphy, Ivdity Chikovani, Nino Makhashvili, Vikram Patel, Martin McKee

**Affiliations:** 1 European Centre on Health of Societies in Transition (ECOHOST), London School of Hygiene and Tropical Medicine, London, United Kingdom; 2 Curatio International Foundation, Tbilisi, Georgia; 3 Global Initiative on Psychiatry – Tbilisi, Georgia, Ilia State University, Tbilisi, Georgia; 4 Centre for Global Mental Health, London School of Hygiene and Tropical Medicine, London, United Kingdom; Max Planck Institute of Psychiatry, Germany

## Abstract

**Background:**

The evidence on alcohol use disorder among conflict-affected civilian populations remains extremely weak, despite a number of potential risk-factors. The aim of this study is to examine patterns of alcohol use disorder among conflict-affected persons in the Republic of Georgia.

**Methods:**

A cross-sectional survey of 3600 randomly selected internally displaced persons (IDPs) and former IDPs. Two alcohol use disorder outcomes were measured: (i) having at least hazardous alcohol use (AUDIT score ≥8); (ii) episodic heavy drinking (consuming >60 grams of pure alcohol per drinking session at least once a week). Individual level demographic and socio-economic characteristics were also recorded, including mental disorders. Community level alcohol environment characteristics relating to alcohol availability, marketing and pricing were recorded in the respondents' communities and a factor analysis conducted to produce a summary alcohol environment factor score. Logistic regression analyses examined associations between individual and community level factors with the alcohol use disorder outcomes (among men only).

**Results:**

Of the total sample, 71% of men and 16% of women were current drinkers. Of the current drinkers (N = 1386), 28% of men and 1% of women were classified as having at least hazardous alcohol use; and 12% of men and 2% of women as episodic heavy drinkers. Individual characteristics significantly associated with both outcomes were age and experiencing a serious injury, while cumulative trauma events and depression were also associated with having at least hazardous alcohol use. For the community level analysis, a one unit increase in the alcohol environment factor was associated with a 1.27 fold increase in episodic heavy drinking among men (no significant association with hazardous alcohol use).

**Conclusion:**

The findings suggest potential synergies for treatment responses for alcohol use disorder and depression among conflict-affected populations in Georgia, as well as the need for stronger alcohol control policies in Georgia.

## Introduction

There is a substantial body of evidence of the high prevalence of mental health disorders among populations affected by armed conflict, particularly post-traumatic stress disorder (PTSD), depression and anxiety [1], [2], [3]. However, concerns have also been expressed about alcohol use disorder amongst these populations and humanitarian guidelines have noted the need to measure and address alcohol use disorder [4], [5], [6], [7], [8].

There are a number of reasons why alcohol use disorder may be a concern among conflict-affected civilian populations. First, they are often exposed to high levels of violent and traumatic events that are strongly associated with mental disorders such as PTSD, depression and anxiety [2], [3], [9]. Both exposure to traumatic events and these mental disorders are in turn associated with alcohol use disorder, with alcohol used as a form of self-medication to ameliorate symptoms of these disorders [10], [11], [12], [13], [14]. Second, armed conflict and related forced displacement of refugees and internally displaced persons (IDPs), commonly lead to worse living conditions, impoverishment, and the loss of family, friends, assets, livelihoods, and self-esteem, and cultural and social support [15]. Alcohol may act as a coping strategy in response to these stressors [5], [16], [17], [18], [19]. Third, alcohol use disorder is an important cause of non-communicable diseases (NCDs) such as cardiovascular disease, cirrhosis, alcohol hepatitis, and diabetes which are of increasing significance among conflict-affected populations [20], [21]. Fourth, alcohol use disorder has behavioural and social impacts, including as a contributor to gender-based violence, which is a major concern in many settings affected by armed conflict [22], [23], [24], [25], [26].

However, while there is a body of research on alcohol use disorder among former military combatants [27], two systematic reviews on alcohol use among conflict-affected civilian populations noted very few studies, particularly in low and middle income countries where the vast majority of conflict-affected populations live [28], [29]. Those studies that have been done often suffer from weak sampling designs, limited statistical analysis, and limited use of standardised and validated measures to assess hazardous alcohol use. In addition, this existing evidence is restricted to individual-level risk-factors, principally gender, age, education, trauma exposure, and mental disorders [28], [29]. To the best of our knowledge, there have been no studies examining the influence of community level factors such as the availability, marketing and pricing of alcohol products. This is despite evidence from stable settings on their influence on alcohol consumption [30], including studies of alcohol outlet accessibility [31], [32], [33] and advertising [34].

For these reasons, there is a need for better evidence on the patterns of alcohol use and its key-risk factors among conflict-affected civilian populations. The overall aim of this study is to examine the patterns of alcohol use disorder among conflict-affected persons in the Republic of Georgia. The specific objectives are to: (i) measure levels of alcohol use disorder; (ii) identify individual-level risk-factors for alcohol use disorder; (iii) identify community level risk-factors for alcohol use disorder.

The Republic of Georgia has been marked by two main phases of conflict, each involving secessionist movements. The first was in the early 1990s, in the regions of Abkhazia and South Ossetia, with fighting leading to the internal displacement of approximately 300,000 people, of whom approximately 200,000 remain as IDPs. The second was in August 2008, when conflict broke out between Georgia and the Russian Federation over South Ossetia, leading to at least 128,000 ethnic Georgians being internally displaced, of which up to 100,000 have now returned to their home areas on the border with South Ossetia [35]. The majority of current IDPs live in government-established IDP settlements/villages while some remain in makeshift ‘collective centers’ in former hotels, schools, factories, and hospitals (particularly those displaced from the 1990s conflicts). IDP communities are characterised by poor living conditions, high unemployment, poverty, limited integration with local communities, and low access to health care [36]. The living conditions and economic prospects of many of the former IDPs from the 2008 conflict who have returned to their home villages in the border region with South Ossetia also appear to be poor, with high unemployment rates and limited access to basic services and amenities [37]. We are aware of only one study that has measured alcohol use among IDPs in Georgia and this was limited to basic descriptions of frequency of alcohol consumption and amount based on a standard drink (undefined) [38].

## Methods and Materials

### Individual level data

The study used a cross-sectional household survey design and multi-stage random sampling, with stratification by region and displacement status. A total sample size of 3600 men and women aged 18 years and over was determined to meet the statistical requirements of the overall study. This consisted of 1200 respondents from each of the 3 main conflict-affected populations in Georgia: those displaced as a result of conflicts in the 1990s (‘1990s IDPs’); those displaced after the 2008 conflict (‘2008 IDPs’); and former 2008 IDPs who have been able to return to their home areas (‘returnees’).

Primary sampling units (N = 360; 120 sampling units per population group) were selected based on probability proportion to size method using a sampling frame of formal and informal IDP settlement population sizes throughout Georgia provided by the Ministry of Internally Displaced Persons, and for ‘returnees’ lists of villages in the border region with South Ossetia provided by the Governor's office in Shida Kartli region. These were considered to be the most accurate lists available. Within each primary sampling unit, households were randomly selected using the random walk method in which involves selecting a random starting direction from a central location in the cluster, with households lying on this transect from the center to the border of the cluster counted and one of them is then chosen at random and the next nearest households subsequently visited [39]. Within the selected household one person (aged ≥18 years) was randomly selected to be interviewed (based on nearest birthday). If that person was not present, a time was arranged for when the surveyor could return to interview them. If there was no response at the household after 3 visits (on different days and at different times), the next household on the route was visited, with the same process used for refusals or interrupted interviews to ensure the desired sample of 3600 respondents. The data collection took place between October and December 2011. All interviews were conducted in Georgian which is the main language used by the study respondents. People with severe intellectual or mental impairment were excluded from the study (N = 10). The questionnaires were interviewer-administered by trained and experienced professional surveyors. In addition to their previous training in survey work, the surveyors received 10 days training focusing on the background and rationale to the study, sampling procedures, mental health surveying and the use of alcohol and mental health outcome measures, trauma exposure questions, inclusion/exclusion criteria, ethical issues and behaviour. The interviews were face-to-face interviews in the respondents' homes. Respondents received a small jar of coffee as compensation for their time. All respondents provided written informed consent prior to their inclusion in the study. Ethics approval for the study was provided by the National Council on Bioethics in Georgia and the Ethics Committee of the London School of Hygiene and Tropical Medicine.

There were two main measures of alcohol use disorder. The first was the Alcohol Use Disorders Identification Test (AUDIT) [40], [41], which has been widely validated and also used with conflict-affected populations [42], [43], [44]. It consists of 10 items with a recall period of the previous 1 year, with a total score of range of 0 to 40. AUDIT's suggested cut points were applied (8–14 indicating hazardous drinking, 15–19 indicating harmful drinking, and 20+ indicating dependent drinking). The second measure of alcohol use disorder was ‘episodic heavy drinking’ which is a significant issue among working-age men in the former Soviet Union [45] and is a major driver of mortality in the region [46]. We use the WHO definition of episodic heavy drinking as consuming more than 60 grams of pure alcohol per drinking session in the past seven days [47]. This is equivalent to around 6 standard drinks. For comparison, the National Institute on Alcohol Abuse and Alcoholism (NIAAA) in the United States use the term ‘binge drinking’ which describes a pattern of drinking that brings a person's blood alcohol concentration to 0.08 grams % or above [48] which is roughly equivalent to around 4 standard drinks (depending on the drinker's weight).

We collected consumption data from questions on the respondent's current frequency of alcohol consumption by beverage type (wine, beer, spirits) and quantity of each beverage type drunk per drinking occasion. We converted this to pure alcohol using conversion rates of: 1 litre of beer = 4 cL; 750 g bottle of wine (home-made or industrially made)  = 9 cL; 1 bottle of 0.5 litre of vodka or strong spirits = 21.5 cL.

The household survey also included items on common disorders with conflict-affected populations of PTSD, depression and anxiety. PTSD was measured using the Trauma Screening Questionnaire (TSQ) which consists of 10 items on PTSD symptoms over the past 1 week, with No ( = 0) and Yes ( = 1) responses which are summed to produce an overall score range of 0–10, with TSQ's cut-off of ≥6 used to indicate possible PTSD [49]. Depression was measured using the Patient Health Questionnaire (PHQ-9) which consists of 9 questions on depression symptoms over the last 2 weeks, with item scores summed to produce a total score range of 0–27, with the PHQ-9's suggested cut-off of ≥10 used to indicate at least moderate depression [50]. Anxiety was measured using the Generalised Anxiety Disorder (GAD-7) instrument which consists of 7 questions on anxiety symptoms over the last 2 weeks, which produces a total score range of 0–21, with the GAD-7's suggested cut-off of ≥10 used to indicate at least moderate anxiety [51]. The psychometric instruments were translated into Georgian for the purposes of the study using standard procedures involving: (i) translation from English into Georgian using professional translators, with translations reviewed by Georgian mental health experts individually and then as a group for cultural relevance, content and concept consistency, clarity and understanding; (ii) a back-translation to check for accuracy, consistency and equivalence, with adjustments made accordingly; and (iii) piloting and field testing to refine the instruments further [52], [53]. The psychometric instruments showed good internal reliability, with Cronbach's alpha scores for the TSQ, PHQ-9, and GAD-7 of 0.86, 0.86, 0.90, and 0.91 respectively. Intraclass correlations for test-retest reliability were 0.97, 0.98, and 0.96 (conducted in a separate pilot survey of 110 randomly selected IDPs living in Tbilisi). For construct validity, the PHQ-9 and GAD-7 each had a single eigenvalue of >1 indicating a single construct, while TSQ showed 2 eigenvalues >1 which related to the two constructs in TSQ of re-experiencing and arousal. The findings also showed good known groups validity and inter-instrument correlation validity and these are detailed elsewhere [54].

The household survey questionnaire also contained items on exposure to a range of violent and traumatic events adapted from the Harvard Trauma Questionnaire [55]. A range of demographic and socio-economic characteristics were also recorded, including: gender, age, education level, current smoking status (yes/no), employment status, household economic situation, and displacement status (1990s IDPs, 2008 IDPs, returnees).

### Community level data

In addition to conducting the household survey, the data collectors also profiled all the 360 primary sampling units used for the household survey. Characteristics of the communities in which the survey respondents were living were systematically recorded through structured observations of the alcohol environment (e.g. prevalence of alcohol advertisements, retail shops selling alcohol, and alcohol prices). The data collectors would locate the center of the selected community as a starting point and follow the random walk method to assess community conditions (travelling approximately 1 km in total). This was done using a ‘Community Observation Form’ (see Materials S1). The community observation form was adapted from the Environmental Profile of a Community's Health (EPOCH), an instrument developed by Chow et al. for the Prospective Urban Rural Epidemiology study (PURE) [56], and subsequently adapted for use in the former Soviet Union [57], [58].

### Individual level analysis

We described sample characteristics, the prevalence of current alcohol use, frequency of drinking, and mean volume of pure alcohol consumed per year for current drinkers by beverage type and in total. The prevalence of episodic heavy drinking for current drinkers was analysed (i.e. ≥60 grams pure alcohol per drinking session at least once a week [47]), along with the mean amount of pure alcohol (grams) consumed per episode of episodic heavy drinking. The prevalence of AUDIT scores was calculated for categories of: no alcohol problem (score <8); hazardous drinking requiring advice (score 8–15); harmful drinking requiring counselling (score 16–19); and dependent drinking requiring treatment (AUDIT score 20+) [40], [41]. These descriptive results were separated by gender given the wide differences in consumption of alcohol between women and men in Georgia.

We then conducted a multivariate regression analysis of individual level characteristics associated with binary outcomes of at least hazardous alcohol use (AUDIT score ≥8) and episodic heavy drinking. This was among men only as the very small number of women precluded any meaningful analysis of the role of gender. Independent variables included in the regression analyses were age, educational attainment, marital status, household economic status, employment status, tobacco use (whether a current smoker or not), displacement status and mental disorders of PTSD, depression and anxiety; and these were selected based on prior evidence indicating possible relationships with alcohol use disorder use among conflict-affected populations [28], [29]. The variables which showed a significant association (*P*<0.05) with the outcomes in bivariate analysis were then entered into a multivariate model in order to adjust for the influence of the other included variables.

All data were weighted to reflect the actual proportions of ‘1990s IDPs’, ‘2008 IDPs’ and ‘returnees’ in the overall conflict-affected population of Georgia, based upon the sampling frames noted above. Data were also adjusted for the cluster survey design. The intra-cluster correlation (ICC) and the design effect (DEFF) from the cluster sampling design for the two main outcomes were ICC 0.06 and DEFF 1.16 for episodic heavy drinking, and ICC 0.14 and DEFF 1.41 for hazardous alcohol use (AUDIT score ≥8). All data were analysed using Stata 12.

### Community level analysis

A key element of assessing the community influences on alcohol use is that different aspects of the environment act in concert [59], so that multiple predictors must be analysed together [60], thereby necessitating the development of a comprehensive approach to assessing the environment influences [61], [62]. In order to do this, we conducted an exploratory factor analysis, using the following variables from the community observations: whether or not alcohol is formally available 24 h/day in the community; density of alcohol outlets in the community; frequency of exterior advertisements for beer, wine and spirits; and the sum of the cheapest cost of 1 L bottles of vodka, wine and beer (we used the sum of the cheapest costs recorded across each community). Using the six measured variables, we conducted the exploratory factor analysis to identify any unmeasured latent factor that could explain the relationship between them, and assigned scores based on this factor. We then used a population averaged regression model to estimate the association between the factor and both episodic heavy drinking and hazardous drinking (as measured by AUDIT), adjusting for potential confounders (age, marital status, education, household economic status, current displacement status and exposure to traumatic events). We conducted this analysis among men only (as only 2% of women in our sample were classified as episodic heavy drinkers). We applied a population average model which uses a generalised estimating equation approach and allows us to estimate of the odds ratios of our outcomes associated with a unit change in our alcohol environment factor across all communities (i.e. comparing two individuals taken from the whole population, irrespective of community), as opposed to within a given community as with random effects models [63]. Our model for this analysis accounts for the correlation between respondents in the same community (cluster); its results therefore apply only to the respondents in our study, and are not representative of all IDPs and returnees in Georgia.

## Results

The response rate for the household survey was 79%. The regions of Georgia that the 3600 respondents were living in were Shida Kartli (39% of respondents), Tbilisi (21%), Samegrelo-Zemo Svaneti (15%), Imereti (9%), Mtskheta-Mtianeti (6%), Kvemo Kartli (3%), Adjara (1%), Kakheti (1%), Racha-Lechkhumi and Kvemo Svaneti (1%); Samtskhe-Javakheti (1%), and Guria (<1%); reflecting the distribution of IDPs living in formal and informal settlements and returnees in Georgia. Around half (48%) of respondents lived in rural locations. The sample characteristics of the 3600 respondents are shown in Table 1. Only around a third of the sample (35%) were men and this reflects findings of other studies of the general population in Georgia as many men have left to find employment elsewhere [64]. The mean ages were 47.1 years for men and 49.39 years for women. Over half of men and women considered their household economic status as bad or very bad, with 34% of men and 22% of women reported being unemployed. The most common traumatic experiences during the conflict and displacement were: lack of shelter; serious injury; being caught directly in combat situation; and experiencing murder, violence acts against family/friends. Mental disorders were significantly higher among women than men, with PTSD being the most prevalent. The sample characteristics by displacement status are provided in Materials S2. Key differences between the 3 groups (1990s IDPs, 2008 IDPs, and returnees) include settlement type, and significantly higher levels of unemployment, trauma exposure (experiencing serious injury) and mental disorders among 1990s IDPs; and these differences are examined in-depth elsewhere [54].

**Table 1:** Total sample characteristics, by gender (N = 3600).

	Men (N = 1248)		Women (N = 2352)	
	N	(%)	N	(%)
**Age:**				
18–29 years	262	(20.96)	383	(16.27)
30–39 years	196	(15.73)	407	(17.31)
40–49 years	215	(17.26)	393	(16.71)
50–59 years	225	(18.05)	421	(17.88)
65+ years	349	(28.00)	749	(31.84)
**Marital status:**				
Married	816	(65.37)	1307	(55.64)
Single	315	(25.25)	301	(12.83)
Divorced	32	(2.56)	129	(5.48)
Widowed	85	(6.81)	612	(26.05)
**Education status:**				
Completed higher education	253	(20.27)	507	(21.57)
Completed secondary school	872	(69.87)	1633	(69.48)
Primary/incomplete secondary	123	(9.86)	210	(8.95)
**Employment status:**				
Unemployed	420	(33.71)	511	(21.74)
Not employed & not seeking work	120	(9.66)	234	(9.95)
In full-time regular work	191	(15.35)	227	(9.64)
In irregular paid work	58	(4.66)	35	(1.51)
Self-employed	60	(4.84)	74	(3.15)
Housewife			446	(19.00)
Subsistence farmer	92	(7.40)	89	(3.78)
Retired	277	(22.23)	686	(29.21)
Other	27	(2.14)	47	(2.02)
**Household economic status:**				
Very good	7	(0.59)	5	(0.21)
Good	23	(1.86)	33	(1.39)
Average	551	(44.19)	1032	(43.89)
Bad	460	(36.85)	850	(36.15)
Very bad	206	(16.50)	432	(18.37)
**Trauma exposure:**				
Lack of shelter	651	(52.17)	918	(39.03)
Serious injury	309	(24.74)	334	(14.22)
Directly in combat situation	635	(50.87)	993	(42.22)
Physical abuse	29	(2.30)	67	(2.85)
Sexual abuse	5	(0.43)	1	(0.06)
Being abducted	32	(2.59)	18	(0.78)
Been tortured	36	(2.85)	17	(0.71)
Experienced murder, violence acts against family/friends	310	(24.81)	514	(21.86)
Witnessed murder, violence acts against stranger	129	(10.36)	132	(5.59)
**Mental disorders:**				
PTSD	235	(19.16)	612	(26.36)
Depression	142	(11.40)	377	(16.02)
Anxiety	96	(7.66)	299	(12.71)

The patterns of alcohol consumption and behaviour are shown in Table 2. 71% of men were current drinkers, compared with 16% of women. 14% of men drank more than once a week, compared to less than 1% of women. Wine was the most frequently consumed alcoholic beverage (53% men and women) which reflects the fact Georgia is a wine producing nation and it is relatively heavily consumed there. Wine was followed by spirits (26% men; 29% women) and then beer (21% men; 17% women). The volume of pure alcohol consumption per year was considerably higher among current drinking men (13.12 L) compared to current drinking women (1.85 L). Of the current drinkers, 12% of the men and 2% of the women were classified as episodic heavy drinking; and 28% of men and 1% of women reported hazardous alcohol use or more serious alcohol use disorders (AUDIT score ≥8).

**Table 2:** Alcohol consumption and behaviour, by gender.

	Men (N = 1248)				Women (N = 2352)			
	N	%	[95% CI]		N	%	[95% CI]	
**Frequency of drinking:**								
Everyday	22	(1.75)	[1.01;	2.49]	3	(0.11)	[0.00;	0.21]
≥4 times a week	24	(1.89)	[1.13;	2.65]	3	(0.11)	[0.00;	0.22]
2–3 times a week	129	(10.32)	[8.61;	12.02]	13	(0.54)	[0.20;	0.87]
2–4 times a month	286	(22.91)	[20.55;	25.28]	42	(1.79)	[1.19;	2.38]
Once a month or less	443	(35.48)	[32.79;	38.18]	422	(17.94)	[16.18;	19.71]
Never	345	(27.64)	[25.23;	30.06]	1870	(79.52)	[78.09;	80.95]
**Mean volume of pure alcohol consumed (litres) per year, by type (current drinkers only):**								
Wine	903	(6.99)	[6.22;	7.74]	483	(0.97)	[0.66;	1.28]
Spirits	902	(3.36)	[2.81;	3.90]	483	(0.54)	[0.33;	0.75]
Beer	901	(2.77)	[2.34;	3.18]	482	(0.32)	[0.21;	0.42]
All 3 types combined	901	(13.12)	[11.80;	14.44]	482	(1.83)	[1.36;	2.31]
**Alcohol use disorder – episodic heavy drinking (current drinkers only):** 1								
% with episodic heavy drinking	110	(12.20)	[10.06;	14.34]	9	(1.86)	[0.06;	3.07]
Mean amount drunk per episode by episodic heavy drinking (pure alcohol grams)	110	(361.89)	[331.97;	391.82]	9	(297.14)	[185.35;	408.83]
**Prevalence (%) of alcohol use disorder – AUDIT (current drinkers only):**								
No alcohol problem (AUDIT score <8)	652	(72.18)	[69.27;	75.11]	478	(98.96)	[98.05;	99.87]
Hazardous drinking – advice required (AUDIT score 8–15)	232	(25.70)	[22.84;	28.55]	5	(1.04)	[0;	1.97]
Harmful drinking – counselling suggested (AUDIT score 16–19)	9	(0.98)	[0.34;	1.63]	0	(0)	[0;	0]
Dependent drinking – treatment required (AUDIT score 20+)	10	(1.13)	[0.45;	1.82]	0	(0)	[0;	0]

CI, confidence interval.

1 Consuming >60 grams of pure alcohol per drinking session at least once a week.

The individual level characteristics associated with alcohol use disorder among men in the multivariate regression analysis are show in Table 3. Those aged 65 and above were less likely to have hazardous alcohol consumption (AUDIT ≥8), while those aged 30–49 were more likely to engage in episodic heavy drinking compared to 18–29 years. Of the individual trauma events, experiencing a serious injury showed a significant association with episodic heavy drinking (OR 2.36) and hazardous alcohol use (OR 1.66). Cumulative trauma events showed an association with hazardous alcohol consumption, but not with episodic heavy drinking. PTSD did not show an association with either episodic heavy drinking or hazardous alcohol use. However, depression was associated with hazardous alcohol use (OR 2.65). There was a mild but significant dose-response relationship between depression and hazardous alcohol use, with a 1 unit increase in the continuous depression score (range 0 to 27) associated with a β 0.20 ([95% CI 0.089; 0.31], P<0.001) increase in the continuous AUDIT score (range 0 to 40). There was no significant association of household economic situation or employment status or tobacco use with either alcohol use disorder outcomes.

**Table 3:** Individual level factors associated with alcohol use disorder (current drinking men only, N = 912).

	Hazardous drinking 1						Episodic heavy drinking 2					
	N	(%)	OR^3^	95% CI		P	N	(%)	OR^3^	95% CI		P
**Age:**												
18–29 years	44	(22.88)	Ref				15	(7.83)	Ref			
30–39 years	55	(36.88)	1.29	[0.63;	2.62]	0.48	29	(19.00)	2.60	[1.21;	5.57]	0.01
40–49 years	74	(38.72)	1.77	[0.90;	3.47]	0.10	35	(18.29)	2.34	[1.12;	4.85]	0.02
50–59 years	46	(27.76)	0.85	[0.41;	1.73]	0.65	14	(8.54)	1.01	[0.44;	2.33]	0.98
65+ years	32	(15.61)	0.16	[0.05;	0.50]	0.00	17	(8.81)	1.02	[0.45;	2.34]	0.96
**Experienced serious injury:**												
Did not experience	214	(26.08)	Ref				73	(10.53)	Ref			
Experienced	34	(41.46)	2.36	[1.38;	4.05]	0.00	36	(17.16)	1.66	[1.00;	2.75]	0.05
**Cumulative Trauma Exposure:**												
0 events	27	(18.23)	Ref						-			
1 event	49	(22.67)	1.40	[0.61;	3.22]	0.43			-			
2 events	54	(30.53)	2.63	[1.17;	5.92]	0.02			-			
3 events	41	(34.10)	2.67	[1.07;	6.67]	0.04			-			
4+ events	78	(32.47)	2.73	[1.22;	6.09]	0.01			-			
**Depression:** 4												
No symptoms	222	(26.60)	Ref						-			
Symptoms	29	(42.15)	2.65	[1.22;	5.76]	0.01			-			

CI, confidence Interval; OR, odds ratios.

1 AUDIT score ≥8

2 Consuming >60 grams of pure alcohol per drinking session at least once a week.

3 OR adjusted for significant (P<0.05) independent variables shown in the table.

4 PHQ-9 score ≥10

- Non-significant association with outcome of episodic heavy drinking.

The community level alcohol characteristics for the study clusters are shown in Table 4. There was a mean of 13.25 alcohol outlets per community, and a greater mean number of beer advertisements per community (1.69) compared to spirits (0.42) and wine (0.12) (reflecting the increased marketing of beer in the region and dominance of home-produced wine in Georgia). The mean minimum price (US$) was 3.63 for 1 L of vodka, 1.51 for 1 L of beer, and 2.35 for 1 L of wine. Over 15% of communities had alcohol available for sale 24 hours a day. The factor analysis revealed only one factor with an eigenvalue of greater than 1 (eigenvalue  = 2.39), which explained 40% of the variance. The factor loadings showing the correlation of each variable with the factor are given in Table 4, with alcohol outlets and wine, beer and spirit advertisements contributing most strongly to the factor.

**Table 4:** Community alcohol characteristics and factor loadings for each characteristic pertaining to the alcohol environment.

	Mean number per community [95% CI]			Factor loading
Alcohol outlets	13.25	[12.78;	13.73]	0.6009
Spirit advertisements	0.42	[0.39;	0.46]	0.8264
Wine advertisements	0.12	[0.10;	0.13]	0.6226
Beer advertisements	1.69	[1.61;	1.77]	0.7843
Alcohol formally available 24 hours a day	16.1% 1	[14.9;	17.3]	0.5697
Sum of cheapest mean cost of beer, wine and spirits (US $) *(based on mean prices (US$) for 1L of vodka ($3.63), beer ($1.51), and wine ($2.35)*	7.61	[7.52;	7.70]	0.1417

CI, confidence interval

For original variables, see Materials S1

1 Proportion of all communities where alcohol was available for sale 24 hours

Our analysis of community factors associated with episodic heavy drinking were restricted to men, as only 2% of women in our sample were classified as episodic heavy drinkers. The results of the population average model for episodic heavy drinking are presented in Table 5 and are adjusted for age and education which were the only two covariates that showed a significant association with episodic heavy drinking in the community level model. The results show that each unit increase in the alcohol environment (alcogenic) factor was associated with a 1.27 fold increase in episodic heavy drinking among men in our study clusters. In other words, a man in a study community with a one unit higher factor score had 1.27 times the odds of having alcohol disorder than an individual in a community one unit lower on the factor score scale. There was no significant association of between the alcohol environment factor and hazardous alcohol use.

**Table 5:** Population average logistic regression model on community level alcogenic factor influence on episodic heavy drinking (current drinking men only, N = 912).

	Episodic heavy drinking 1			
	OR^2^	S.E	[95% CI]	P
				
**Alcogenic factor** 3	**1.27**	**0.15**	**[1.01; 1.59]**	**0.04**
				
**Age category:**				
18–29	Ref			
30–39	3.13	1.53	[1.20; 8.17]	0.02
40–49	2.67	1.15	[1.14; 6.23]	0.02
50–59	1.60	0.82	[0.59; 4.38]	0.36
60+	0.58	0.38	[0.16; 2.06]	0.40
**Education:**				
No education/primary/incomplete secondary	Ref			
Complete secondary/incomplete higher	0.38	0.18	[0.15; 0.97]	0.04
Complete higher	0.29	0.17	[0.09; 0.91]	0.03

CI, confidence interval; OR, odds ratio; S.E, standard error (robust).

1 Episodic heavy drinking classified as consuming 60 grams of pure alcohol per drinking session at least once a week.

2 Odds ratio adjusted for age and education. It represents increased odds of episodic heavy drinking for each unit increase in the alcohol environment (alcogenic) factor score).

3 Alcogenic factor comprising a single factor from variables on alcohol outlets, spirits/wine/beer advertising, alcohol formally available 24 hours a day, and sum of cheapest mean cost of beer, wine and spirits.

## Discussion

This study presents data on individual and community level influences on alcohol use disorder among conflict-affected persons in Georgia. To the best of our knowledge, it is the first study to examine the role of community level alcohol-related factors on alcohol use disorder among conflict-affected civilian populations.

The findings suggest that the volume of alcohol consumed appears to be slightly lower for men in our study than reported by WHO for the general male population in Georgia (13.12 L our study; and 14.81 WHO (equivalent to around 200 grams/20 standard drinks per week)) and substantially lower for women (1.85 L our study; 9.44 L WHO) [23]. The WHO do not, however, indicate the source of their data, although often it is derived from trade balance data, and the WHO data on consumption recorded among women does seem rather high. Significantly lower alcohol consumption among women in Georgia is also shown in other studies [38], [65]. The large gender difference in our data may be partly attributable to conflict-affected women coming from rural areas where drinking among women is generally lower than in urban areas in Georgia. The proportion of episodic heavy drinking among current drinkers was also lower in our study than reported by WHO for the general Georgian population, both for men (our study 12.2%; WHO 27.3%) and women (our study 1.86; WHO 4.0%). Overall, these findings are consistent with those from other studies indicating that exposure to armed conflict and forced displacement have not resulted in increased levels of alcohol consumption for the overall conflict-affected population when compared to the non-conflict-affected populations in the same country [42], [43], but that consumption and alcohol use disorder may be high among certain groups.

The odds of episodic heavy drinking and hazardous drinking were greatest at ages 30–49 years when compared with the 18–29 year old group. This could possibly relate to higher levels of exposure to violent and traumatic events and broader dissatisfaction with the effects of conflict and displacement in the age group 30–49 years. However, these findings are similar to those from earlier research with the general population in Georgia in which 30–49 year olds were more likely to have heavy drinking situations [66]. It seems that while patterns of episodic heavy drinking are commonly higher in younger age groups globally (particularly in high-income settings), such patterns are not yet present in Georgia (both for conflict-affected and general populations) and nor are they present in most other countries in the former Soviet Union [45]. Evidence from other studies on conflict-affected civilian populations on the influence of age is mixed, with younger age associated with hazardous alcohol use in two studies of South East Asian refugees in the United States [17], [19], while a study of IDPs in northern Uganda observed higher levels among older age groups [43], and a study of Bhutanese refugees in Nepal observed no age effect [42]. Our study found no association of level of education with alcohol use disorder at the individual level (but there was in the community level analysis); and a study on harmful alcohol use among Bhutanese refugees in Nepal did observe an association between higher education and lower alcohol use disorder [42]. No association was observed between tobacco use and alcohol use disorder (for further details on tobacco use with the study population please see [67]), whereas the study of Bhutanese refugees in Nepal did report an association between tobacco use and hazardous alcohol use [42].

Our study indicated an association of cumulative trauma exposure with hazardous drinking, but not with episodic heavy drinking. Of the individual trauma variables, experiencing a serious injury was associated with both hazardous alcohol use and episodic heavy drinking. Findings from other studies among conflict-affected civilian populations have also shown associations between problematic drinking and exposure to violent and traumatic events, particularly cumulative trauma exposure [17], [43], [68].

PTSD was not associated with either alcohol outcome but depression was associated with hazardous alcohol use. This is consistent with studies from stable settings on the association of alcohol use and common mental disorders, particularly depression [11], [69], [70], [71]. Establishing the causal pathways involved is complex and beyond the scope of this paper. However, studies from stable settings have indicated a number of ways in which alcohol use disorder can increase the risk of mental disorders, such as: negatively impacting on individual's socio-economic circumstances which may then lead to worse mental health; alcohol and mental disorders being linked by genetic factors relating to neurotransmitter functioning which increase the risk of mental disorders in the presence of heavy alcohol use; and alcohol use causing metabolic changes that increase the risk of mental disorders [72], [73], [74]. Other studies suggest that individuals with poor mental health are more prone to use alcohol as a negative form of coping with the effects of the symptoms of mental disorders [75], [76], [77]. Given the association between common mental disorders and alcohol use disorder, including how individuals with mental disorders may also use alcohol to self-medicate the mental disorder symptoms [77], a combined approach to treatment may yield the greatest benefits for those presenting with both disorders. However, mental health services remain generally limited in Georgia, including with the conflict-affected population [78], [79]. Services for alcohol use disorder also are extremely limited, and integrated mental and alcohol disorder services appear rare [80]. Further research is required with conflict-affected populations to better understand the causal pathways between alcohol use disorder and common mental disorders. In addition, studies are required to assess the effectiveness of alcohol interventions with conflict-affected civilian populations in low and middle income settings as evidence on this appears limited to a single study [81].

Our findings also suggest that at the community level alcohol-related environmental characteristics combine to increase episodic heavy drinking in our study clusters. In our analysis, we found that one underlying factor accounts for the correlation between several measures of the alcohol environment, and that, of the measures included in the factor analysis, the frequency of alcohol advertisements and alcohol outlets contributed most to this factor. In other words, the findings of this analysis suggest that a high number of beer, wine and spirit advertisements, high alcohol outlet density and availability of alcohol may work together to create an ‘alcogenic’ environment that encourages episodic heavy drinking among the respondents in our study. The underlying factor identified in this analysis was not statistically significantly associated with hazardous drinking as defined by the AUDIT questionnaire, which may suggest that community level factors influence volume and frequency of alcohol consumption but not other elements of alcohol use disorder as measured by AUDIT, but further research is required to explain this finding. Although price did not contribute significantly to the factor, this was possibly due to the inability of the community profile instrument to capture the availability and price of home-made alcohols, or the lack of variability in price among communities included in this analysis. Our findings are consistent with those from other multi-level studies conducted in the United States that found a relationship between availability, advertising and alcohol consumption [30], [34], [82], [83]. This implies that alcohol control policies in Georgia also need to address community influences on alcohol use disorder, following international evidence and policy guidance on controlling alcohol availability, marketing and pricing [84], [85], [86]. However, current alcohol control policies in Georgia are weak and there is no national action plan on alcohol. More specifically, there are no restrictions on hours of sale, density of outlets, or marketing of alcohol in the media and advertising portals; alcohol prices are relatively low and there is no alcohol taxation policy aiming to reduce alcohol consumption; and there are no mass media programmes on alcohol education [87].

### Limitations

The cross-sectional study designs means that causation cannot be attributed. There is the potential for reverse causality linking alcohol advertisements and outlets and alcohol consumption (i.e. it is possible that alcohol companies simply increase marketing and availability in communities where there are more drinkers). However, a global review using longitudinal studies found strong and consistent evidence that exposure to advertising increases the likelihood of drinking initiation and consumption [88]. Additional limitations include the small number of women drinkers which meant they could not be meaningfully included in the individual and community level regression models. The sample size may have prevented identification of associations with alcohol use disorder, such as some of the individual trauma exposures. The use of self-reported consumption is also a limitation because of frequent under-reporting [89] and response bias, whereby the heaviest drinkers tend to be underrepresented in surveys as they may not be present, willing or able to participate in such surveys (which may partially explain the lower consumption patterns reported in our survey compared to the WHO estimates). The validity of the AUDIT instrument was not assessed in this study population, but the internal reliability was good (Cronbach's alpha 0.91). The study did not explore any role of family history of alcohol disorder and there are well recognised genetic and parental behavioural influences on alcohol use disorder. The study also did not include use of illicit drugs and their association with alcohol use, and there is some evidence that illicit drug use is an increasing problem in Georgia [90], [91]. Finally, the study did not include IDPs hosted by relatives or friends or living independently away from the formal and informal IDPs settlements.

### Conclusions

Armed conflicts and forced displacement are commonly protracted over years and even decades. Policies, programs and research are needed to address both short-term humanitarian relief and longer-term recovery situations, including damaging health behaviours such as alcohol use disorder. Our study highlights the links between trauma exposure, depression and alcohol use disorder among conflict-affected populations in Georgia. It also indicates an association between combined community level influences of alcohol availability, advertising and pricing with alcohol use disorder. The findings suggest potential synergies for individual level treatment responses for alcohol use disorder and depression among conflict-affected populations in Georgia, as well as the need for stronger population level alcohol control policies in Georgia.

## Supporting Information

Materials S1 **Community Observation Form (alcohol section only).**
(DOC)

Materials S2 **Selected sample characteristics, by displacement status (N = 3600).**
(DOCX)
