# Supplementary material for: Individual and Community Level Risk-Factors for Alcohol Use Disorder among Conflict-Affected Persons in Georgia
Source: PLoS One. 2014 May 27;9(5):e98299. doi: 10.1371/journal.pone.0098299 (PMC4035315; doi:10.1371/journal.pone.0098299)
Supplement: Materials S1 — Community Observation Form (alcohol section only). (DOC) [file pone.0098299.s001.doc]

**Table S1: Community Observation Form (alcohol section only)**

| IDP MENTAL HEALTH PROJECT – COMMUNITY OBSERVATIONSCommunity Observation Form (alcohol section only) |
| --- |

| **Background information** |
| --- |

#### Name of the region: _______________________________

#### Name of the district: _______________________________

#### Name of the settlement: _______________________________

#### Code for primary sampling Unit (same as hh survey PSU): |_____|_____|_____|

(Note: If community observation form covers area which includes several hh survey PSUs, then please also add these additional hh survey PSUs: **|_____|_____|_____|**

#### |_____|_____|_____|

#### |_____|_____|_____|

#### |_____|_____|_____|

#### Is settlement in a rural area? 1=yes, 2 =No

#### If IDP settlement is in rural area, please:

#### Give the name of the nearest large town/city __________________________

#### Approximate the distance to this nearest large town/city _________ KMs

#### Settlement GPS coordinates (to be filled in beforehand or afterwards):

_________________

#### Date:

Day / Month / 2011

#### Time:

hour / minute

| **Alcohol information** |
| --- |

**Notes:**

- **Use centre of the selected community as starting point and follow random walk method to assess the alcohol environment in the community (travelling approximately 1 km in total).**
- **For the questions on access and prices of alcohol, please also go to the nearest commercial area where most people from the community would purchase alcohol from. You can select this area based upon your observations and the locally knowledgeable people you spoke for previous parts. These questions on alcohol should be completed based upon your observations in both the residential and commercial areas if separate.**

#### What are places where you can buy alcohol 24 hours in the day? (Mark all that apply)

|  |  | Yes | No |
| --- | --- | --- | --- |
| A | Shops | 1 | 2 |
| B | Kiosks | 1 | 2 |
| C | Bars/Cafes/ Restaurants | 1 | 2 |
| D | A person on the street | 1 | 2 |
| E | Someone’s private house | 1 | 2 |

#### Count the number of advertisement for alcohol (any type) you see and mark down in each category. Advertisements include those on billboards, pasted on shop windows, bus shelters or other locations that is easily visible from the street.

| **Advertisement/ sign** | **Tally** | **Total number** |
| --- | --- | --- |
| 1. Alcohol (wine) |  |  |
| 1. Alcohol (beer) |  |  |
| 1. Alcohol (spirits) |  |  |

#### Count the number of outlets you see that sell alcohol and mark down in each category.

| **Outlets** | **i. Super market** | **ii.Alcohol shop** | **iii.Market -place** | **iv.Small shop** | **v. Kiosk** | **vi. Bar/Café/**  **restaurant** | **vii. Total number** |
| --- | --- | --- | --- | --- | --- | --- | --- |
| - - - 1. Wine |  |  |  |  |  |  |  |
| B. Beer |  |  |  |  |  |  |  |
| C. Spirits |  |  |  |  |  |  |  |

**Collect the costs of the following items. If amount is different to 1 litre, please recalculate cost as equivalent for 1 litre.**

Store types codes:

| Supermarket = 1 | Kiosk=5 |
| --- | --- |
| Alcohol shop = 2 | Bar / Café/restaurant =6 |
| Market-place = 3 |  |
| Small shop = 4 | Other=7 |

|  | **Product** | **Cost of cheapest item** | **Code of Store at which cost obtained** |
| --- | --- | --- | --- |
| A | Beer (1 litre) |  |  |
| B | Wine (1 litre) |  |  |
| C | Vodka (1 litre) |  |  |
| D | Home-made wine (1 litre) |  |  |
| E | Home-made vodka (1 litre) |  |  |
