# Supplementary material for: Individual and Community Level Risk-Factors for Alcohol Use Disorder among Conflict-Affected Persons in Georgia
Source: PLoS One. 2014 May 27;9(5):e98299. doi: 10.1371/journal.pone.0098299 (PMC4035315; doi:10.1371/journal.pone.0098299)
Supplement: Materials S2 — Selected sample characteristics, by displacement status (N = 3600). (DOCX) [file pone.0098299.s002.docx]

**Table S2: Selected sample characteristics, by displacement status (N=3600)**

|  | **1990s IDPs (N=1200)** | | | **2008 IDPs (N=1200)** | | | **Returnees (N=1200)** | | |
| --- | --- | --- | --- | --- | --- | --- | --- | --- | --- |
|  | **%** | **[95% CI]** | | **%** | **[95% CI]** | | **%** | **[95% CI]** | |
| **Gender:** |  |  |  |  |  |  |  |  |  |
| Men | 34.67 | [31.97; | 37.36] | 33.42 | [30.74; | 36.09] | 35.92 | [33.20; | 38.63] |
| Women | 65.33 | [62.64; | 68.03] | 66.58 | [63.91; | 69.26] | 64.08 | [61.37; | 66.80] |
| **Age:** |  |  |  |  |  |  |  |  |  |
| 18-29 years | 18.92 | [16.70; | 21.14] | 20.17 | [17.89; | 22.44] | 15.58 | [13.53; | 17.64] |
| 30-39 years | 17.83 | [15.66; | 20.00] | 19.50 | [17.26; | 21.74] | 14.17 | [12.19; | 16.14] |
| 40-49 years | 16.25 | [14.16; | 18.34] | 17.50 | [15.35; | 19.65] | 17.83 | [15.66; | 20.00] |
| 50-59 years | 18.75 | [16.54; | 20.96] | 15.75 | [13.69; | 17.81] | 17.17 | [15.03; | 19.30] |
| 65+ years | 28.25 | [25.70; | 30.80] | 27.08 | [24.57; | 29.60] | 35.25 | [32.54; | 37.96] |
| **Education:** |  |  |  |  |  |  |  |  |  |
| Completed higher education | 25.17 | [22.71; | 27.63] | 20.78 | [18.48; | 23.09] | 14.33 | [12.35; | 16.32] |
| Completed secondary school | 67.67 | [65.02; | 70.32] | 67.20 | [64.53; | 69.86] | 73.58 | [71.09; | 76.08] |
| Primary/incomplete secondary | 7.17 | [5.71; | 8.63] | 12.02 | [10.18; | 13.86] | 12.08 | [10.24; | 13.93] |
| **Settlement type:** |  |  |  |  |  |  |  |  |  |
| New camp settlement | 0.00 | [0.00; | 0.00] | 70.83 | [68.26; | 73.41] | 0.00 | [0.00; | 0.00] |
| Collective centre (old camp/block) | 99.91 | [0.00; | 0.26] | 29.17 | [26.59; | 31.74] | 1.33 | [0.68; | 1.98] |
| Home location | 0.09 | [99.74; | 100.00] | 0.00 | [0.00; | 0.00] | 98.67 | [98.02; | 99.32] |
| **Employment status:** |  |  |  |  |  |  |  |  |  |
| Unemployed | 33.08 | [30.42; | 35.75] | 22.74 | [20.36; | 25.12] | 14.67 | [12.66; | 16.67] |
| Not employed & not seeking work | 9.08 | [7.46; | 10.71] | 8.61 | [7.02; | 10.20] | 11.5 | [9.69; | 13.31] |
| In full-time regular work | 12.17 | [10.31; | 14.02] | 16.47 | [14.37; | 18.58] | 9.42 | [7.76; | 11.07] |
| In irregular paid work | 3.5 | [2.46; | 4.54] | 3.6 | [2.54; | 4.65] | 0.83 | [0.32; | 1.35] |
| Self-employed | 4.17 | [3.03; | 5.30] | 1.09 | [0.50; | 1.68] | 3.75 | [2.67; | 4.83] |
| Housewife | 10.67 | [8.92; | 12.42] | 19.48 | [17.23; | 21.73] | 13.25 | [11.33; | 15.17] |
| Subsistence farmer | 0.25 | [0.00; | 0.53] | 0.25 | [0.00; | 0.53] | 14.5 | [12.50; | 16.50] |
| Retired | 24.58 | [22.14; | 27.02] | 25.17 | [22.70; | 27.63] | 30.92 | [28.30; | 33.54] |
| Other | 2.5 | [1.62; | 3.38] | 2.59 | [1.69; | 3.49] | 1.17 | [0.56; | 1.78] |
| **Household economic status:** | |  |  |  |  |  |  |  |  |
| Very good | 0.33 | [0.01; | 0.66] | 0.42 | [0.05; | 0.78] | 0.33 | [0.01; | 0.66] |
| Good | 1.58 | [0.88; | 2.29] | 2.17 | [1.34; | 2.99] | 1.34 | [0.68; | 1.99] |
| Average | 45.25 | [42.43; | 48.07] | 53.17 | [50.34; | 55.99] | 39.32 | [36.55; | 42.09] |
| Bad | 33.92 | [31.23; | 36.60] | 34.42 | [31.72; | 37.11] | 41.15 | [38.36; | 43.94] |
| Very bad | 18.92 | [16.70; | 21.14] | 9.83 | [8.15; | 11.52] | 17.86 | [15.69; | 20.04] |
| **Trauma:** |  |  |  |  |  |  |  |  |  |
| Experienced serious injury | 21.00 | [18.69; | 23.31] | 15.08 | [13.06; | 17.11] | 13.42 | [11.49; | 15.35] |
| **Mental disorders:** |  |  |  |  |  |  |  |  |  |
| PTSD | 26.93 | [24.39; | 29.47] | 25.25 | [22.78; | 27.73] | 18.26 | [16.05; | 20.46] |
| Depression | 18.67 | [16.46; | 20.87] | 11.75 | [9.93; | 13.57] | 7.92 | [6.39; | 9.45] |
| Anxiety | 13.00 | [11.09; | 14.91] | 10.50 | [8.76; | 12.24] | 7.58 | [6.08; | 9.08] |
